# The Specific Pathogenicity Pattern of the Different *CRB1* Isoforms Conditions Clinical Severity in Inherited Retinal Dystrophies

**DOI:** 10.3390/ijms262311551

**Published:** 2025-11-28

**Authors:** Laura Siles, Sheila Ruiz-Nogales, Pilar Méndez-Vendrell, Anniken Burés-Jelstrup, Rafael Navarro, Esther Pomares

**Affiliations:** 1 Departament de Genètica, Institut de Microcirurgia Ocular, IMO Grupo Miranza, 08035 Barcelona, Spain; laura.siles@imo.es (L.S.); sheila.ruiz@imo.es (S.R.-N.); pilar.mendez@imo.es (P.M.-V.); 2 Departament de Retina, Institut de Microcirurgia Ocular, IMO Grupo Miranza, 08035 Barcelona, Spain; anniken.bures@imo.es (A.B.-J.);

**Keywords:** CRB1, inherited retinal dystrophies, retinal organoids, Leber congenital amaurosis, cone–rod dystrophy, cone dystrophy, macular dystrophy, genotype–phenotype correlation, retinal pigment epithelium, photoreceptors

## Abstract

Pathogenic variants in Crumbs homolog 1 (*CRB1*) cause a wide range of severe ocular diseases, most commonly Leber congenital amaurosis and other forms of adult-onset macular dystrophy that lead to vision loss. Despite this broad clinical spectrum, the expression and function of *CRB1* in retinal cells remains underexplored. In this study, we show a comprehensive characterization of *CRB1* isoforms in several human retinal models like retinal organoids. Although *CRB1* is predominantly expressed in photoreceptors and Müller glial cells, we also detected its expression in the human retinal pigment epithelium (RPE). Moreover, we observed defined expression patterns of *CRB1* isoforms depending on the maturation stage of retinal cells, suggesting a role for this protein in development and differentiation. In this context, the less abundant and less studied isoform *CRB1-C* was the most highly expressed in early undifferentiated stages of photoreceptors and in RPE. Additionally, clinical and genetic evaluation of a cohort of 25 probands carrying pathogenic *CRB1* variants allowed us to propose a genotype–phenotype correlation between isoforms involvement and disease severity, and to the identification of four novel pathogenic variants: p.Met70ArgfsTer17, p.Cys136Phe, p.Cys248Ser and p.Gln1094Ter. Collectively, our data elucidate previously undescribed expression patterns of *CRB1* isoforms during retinal cell differentiation and highlight key aspects of *CRB1*-associated inherited retinal dystrophies.

## 1. Introduction

Inherited retinal dystrophies (IRD) are a heterogeneous group of rare disorders characterized by several visual complications that ultimately lead to vision loss and, in most cases, still lack effective treatments. They are caused by mutations in more than 300 genes essential for retinal cell function, including photoreceptors, the retinal pigment epithelium (RPE), and Müller glial cells [1,2]. Mutations in *Crumbs cell polarity complex 1* (*CRB1*) gene cause several types of IRD and, in Spain, represent the most frequent pathogenic variants among patients with Leber congenital amaurosis (LCA) [3].

In mammals, the CRB family consists of CRB1, CRB2, CRB3A, and CRB3B [4]. The CRB1 protein contains multiple structural domains, including a large extracellular region with epidermal growth factor (EGF) and laminin-globular domains, and an intracellular C-terminus with single FERM and PDZ protein-binding domains [5]. CRB1 is highly expressed in the retina and brain, although it is also present in other tissues like kidney, lung, testis, and stomach [4,6]. In these tissues, the CRB protein complex regulates apical-basal polarity, modulates apical membrane size, and maintains cell adhesion through the cadherin–catenin complex at adherens junctions [7]. Particularly, in the retina, the CRB complex is essential for photoreceptor morphogenesis, and is localized at the junctions between photoreceptors, Müller glial cells, and photoreceptors and Müller glial cells [8,9,10,11]. Specifically, CRB1 is present in the subapical region either above adherens junctions within Müller glial cells microvilli and in the inner segments of the photoreceptors [12]. Beyond this subapical localization, CRB1 is also expressed in the outer plexiform layer of Müller glial cells, surrounding photoreceptor axons within the Henle fiber layer of the fovea [4,13,14].

*CRB1* is located on chromosome 1q31.3 and, to date, three major isoforms have been described to be expressed in the human retina (*CRB1-A*, *CRB1-B*, and *CRB1-C*) [15]. The canonical isoform, *CRB1-A*, consists of 12 exons spanning approximately 210 kb of genomic DNA [6,16]. However, Ray et al. reported that *CRB1-B* is more abundant than *CRB1-A* in both mouse and human retina [15]. In fact, *CRB1-A* is mostly expressed in Müller glia cells, whereas *CRB1-B* is found in rod and cone photoreceptors [15,17]. The *CRB1-B* transcript encodes a transmembrane protein with partial similarity to *CRB1-A* in its extracellular domain but with significant differences in its intracellular region [18]. Interestingly, although *CRB1* has been traditionally associated with photoreceptors and Müller glial cells, recent studies have reported its expression in mouse RPE and iPSC-derived RPE models, although its expression in human cells remains unexplored [19,20]. The third isoform, *CRB1-C*, encodes a 754-amino acid protein lacking transmembrane and intracellular domains, and its expression and function remain largely unknown [21].

Biallelic *CRB1* variants have been associated with multiple IRD-phenotypes; however, to date, there is a lack of defined genotype–phenotype correlations that could help elucidate its role in their pathogenesis. Currently, more than 500 mutations (HGMD professional, 2025.2) have been described associated to a broad spectrum of retinal phenotypes including (I) Leber congenital amaurosis type 8 (LCA8), an early-onset cone–rod retinal dystrophy, (II) cone dystrophy (CD), characterized primarily by cone dysfunction, and (III) cone–rod dystrophy (CRD), in which cones are initially affected followed by rod degeneration [22,23,24,25,26]. CD and CRD typically present later in life compared to LCA [23,27]. Less common phenotypes associated with *CRB1* mutations include Coat’s-like vasculopathy (also known as Coat’s-like retinitis pigmentosa), keratoconus, and nanophthalmos [28].

Over the past years, several groups have attempted to establish genotype–phenotype correlations for *CRB1* mutations to explain IRD clinical heterogeneity. For example, the variant p.C948T in homozygosis was initially associated with LCA but has also been reported in retinitis pigmentosa [29,30,31]. Other recently published studies found no significant correlation between disease progression and domain-specific variant location, although most variants clustered in exons 6, 7, and 9, affecting both *CRB1-A* and *CRB1-B* [20,32,33,34,35].

In this study, we aimed to characterize isoform-specific expression patterns in several retinal cell models and during retinal development using hiPSC-derived cellular models. Interestingly, we found that isoform abundance is strongly dependent on the maturation stage of the tissue and not solely on the retinal cell type. Retinal organoids (ROs), which are 3D models that mimic human retinal structure, exhibited high levels of *CRB1-A* at early stages that decreased as the ROs matured. Additionally, we found that RPE cells displayed *CRB1* expression, although at low levels, at both mRNA and protein levels, and that its localization changed with maturation in culture. Furthermore, our results indicate that *CRB1-C* is indeed expressed during retinal cell differentiation, particularly at early stages. Finally, the analysis of a cohort of 25 patients carrying pathogenic *CRB1* variants revealed a genotype–phenotype correlation and suggested a classification framework for CD, CRD, and LCA based on the impact of isoform expression. This classification may support clinicians in predicting disease prognosis and highlights the need for a deeper understanding of *CRB1* expression and function in retinal cells.

## 2. Results

### 2.1. Differential Expression of CRB1 Isoforms During Retinal Development and Maturation

*CRB1* has traditionally been associated with Müller glial cells and photoreceptors [36]. However, recent studies have provided insights into its expression in other cell-types, such as the RPE, in both murine and human models [19,20]. Despite this, the expression of *CRB1* isoforms in human cells has been only barely explored [15,20]. Thus, we sought to investigate *CRB1* expression across several human retinal models and differentiation stages.

**Figure 1:**
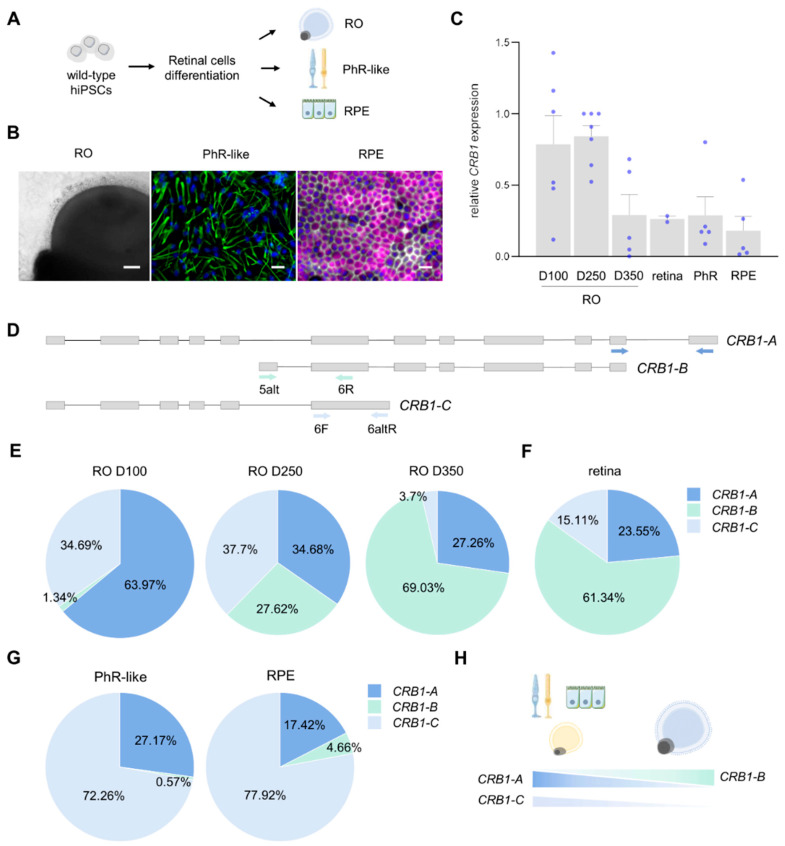
Differential expression of *CRB1* isoforms during retinal cells development and maturation. (**A**) Scheme of the generation of human retinal models. (**B**) Representative pictures of the three models in (**A**). In immunofluorescence captures: green is rhodopsin, grey is ZO-1, magenta is RPE65, and cells were counterstained with DAPI. Scale bar represents 50 µm in RO and 200 µm in PhR-like and RPE. (**C**) Relative mRNA expression of total *CRB1* at different maturation days (‘D’). (**D**) Schematic representation of *CRB1* isoforms where arrows indicate localization of the primer pairs used for quantification. CRB1-A was detected using commercially available Taqman probes as detailed in Section 4. (**E**) Pie chart representation of isoforms expression distribution relative to total *CRB1* in hiPS-RO. The mean of at least four samples is shown. (**F**) As in (**E**) but in retinal cDNA from a human donor. (**G**) As in (**E**) but in PhR-like and RPE cultures. At least three independent samples were used. (**H**) Scheme depicting isoform abundance in the hiPSC-derived retinal models. Abbreviations: RO, retinal organoid; PhR, photoreceptor-like; RPE, retinal pigment epithelium.

We first differentiated wild-type hiPSCs into RO, RPE, and photoreceptor-like (PhR-like) cells, and analyzed the expression of multiple retinal markers together with total *CRB1* (Figure 1A–C and Figure S1A). A commercially available cDNA sample from a 77-year-old human retinal donor was included as a control. Interestingly, hiPS-derived RO showed the highest levels of total *CRB1* expression between days 100 and 250 of maturation (D100-D250), followed by a decrease on day 350 (D350) (Figure 1C). Notably, at D350, total *CRB1* levels were comparable to those found in the human retina donor cDNA, as well as in photoreceptor-like cells and RPE (Figure 1C). Importantly, we detected *CRB1* mRNA in human RPE where its expression has previously been only scarcely analyzed [20].

Next, we used isoform-specific primers to detect all three known *CRB1* isoforms, as illustrated in Figure 1D. In order to compare the relative mRNA expression of the different isoforms, we applied the Pfaffl method [37], a mathematical model that enables accurate comparison of genes in qPCR assays by incorporating the amplification efficiency of each primer pairs used for detecting the *CRB1* isoforms (Figure S1B).

Analysis of mRNA levels of the specific isoforms relative to total *CRB1* in each sample showed that *CRB1-A* was the most abundant isoform at D100 of RO differentiation and progressively decreased as the RO matured (Figure 1E). In contrast, *CRB1-B* expression increased over time, and by D250, the relative proportions of the three isoforms were nearly equivalent (Figure 1E). Notably, D350 RO exhibited an isoform expression pattern highly similar to that found in the human retinal donor cDNA, indicating a strong convergence between these two samples (Figure 1E,F). *CRB1-C* was also substantially expressed on D100 and D250 but was barely detectable at D350, suggesting a specific role during earlier stages of retinal development (Figure 1E and Figure S1C). Moreover, this isoform was the most abundant in photoreceptor-like cells and RPE, which also expressed *CRB1-A* and very low levels of *CRB1-B* (Figure 1G,H and Figure S1C). It is worth noting that photoreceptor-like cells generated in these 2D cultures exhibit a less differentiated and mature photoreceptor phenotype compared to other retinal cell models. Conversely, RO contain mature photoreceptors with well-formed outer segments, as well as other retinal cell types such as Müller glial cells, which may influence isoform expression levels.

**Figure 2:**
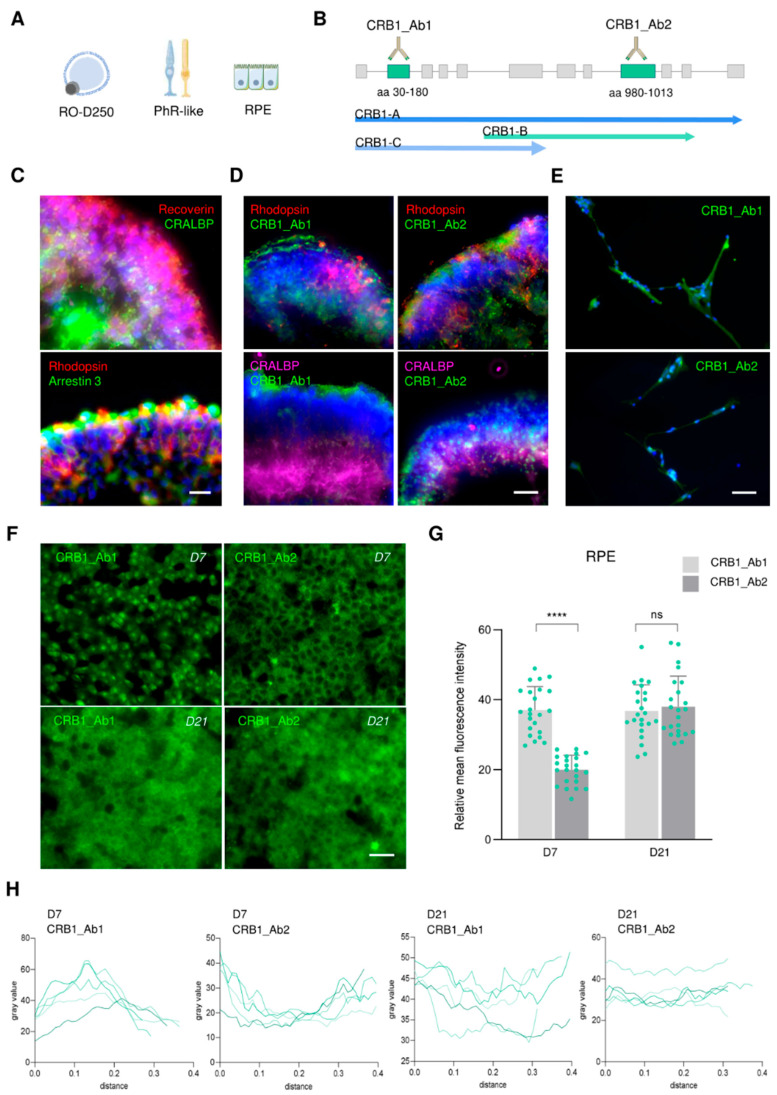
Expression of CRB1 in retinal cells models. (**A**) Human retinal models used for immunofluorescence analysis. (**B**) Antibodies used for detecting CRB1 isoforms raised against amino acids (aa) 30-180 (CRB1_Ab1) or 980-1013 (CRB1_Ab2). (**C**) Immunofluorescence staining of photoreceptors (Recoverin), Müller glia cells (CRALBP), rods (Rhodopsin) and cones (Arrestin 3) in hiPS-RO (D250). Scale bar represents 20 µm. (**D**) Representative pictures of CRB1 staining with Rhodopsin and CRALBP markers in hiPS-RO. Scale bar represents 25 µm. (**E**) Representative captures of CRB1 immunofluorescence in PhR-like cells. Scale bar represents 50 µm. (**F**) As in (**E**) but in RPE on day 7 or day 21 of maturation. Scale bar represents 25 µm. (**G**) Quantification of relative mean fluorescence intensity in (**F**). At least 20 cells were analyzed. (**H**) Plot of the fluorescence intensity values across a cell showing five representative quantifications in (**F**). Statistical significance was assessed with non-parametric Mann–Whitney (*p* ≤ 0.0001 (****) levels, or non-significant (ns) *p* > 0.05).

Next, we evaluated CRB1 expression at the protein level using immunofluorescence. For this, we focused on D250 RO, as they exhibit a mature retinal structure and express high levels of all three isoforms, similar to RPE and photoreceptor-like cells (Figure 2A). We used two commercially available antibodies targeting amino acids 30-180 (recognizing CRB1-A and CRB1-C) and amino acids 980-1013 (detecting CRB1-A and CRB1-B), hereafter referred as CRB1_Ab1 and CRB1_Ab2, respectively (Figure 2B and Table S1). In RO, we combined CRB1 immunostaining with markers specific for photoreceptors and Müller glial cells to distinguish the respective cellular layers (Figure 2C). Notably, the proportion of rods and cones in the hiPS-derived RO generated in this study was comparable, as shown by the pan-rod marker rhodopsin, and the pan-cone marker arrestin 3 (Figure 2C).

Mature D250 RO displayed a balanced distribution of isoforms mRNA expression as observed in Figure 1E. Similarly, we detected positive staining using both antibodies. In both cases, we observed immunofluorescence in the outer layer of the RO corresponding to the photoreceptors, and in Müller glial cells, as confirmed by the co-staining using Rhodopsin and CRALBP antibodies (Figure 2D). Likewise, photoreceptor-like cells were positive using both antibodies, although a stronger staining was observed with CRB1_Ab1, which recognizes CRB1-A and CRB1-C, in line with the results obtained at the mRNA level (Figure 1G and Figure 2E).

The expression of CRB1 in the retinal epithelial barrier has only been described in mice recently, where it was shown to be critical for epithelial cells’ integrity, and in hiPS-RPE by mRNA and Western blotting (Figure 1C,G) [19,20]. Remarkably, we observed positive staining in hiPS-RPE junctions using both CRB1 antibodies in this human model (Figure 2F and Figure S2). Specifically, when using CRB1_Ab1, we found a strong staining at early stages of RPE maturation (cells cultured for 7 days, D7), that was localized at the center of the cell near the nucleus, while CRB1_Ab2 exhibited a slighter staining at the perimeter of the cell (Figure 2F and Figure S2). These results are consistent with the higher proportions of *CRB1-A* and *CRB1-C* mRNA levels detected in this cell type (Figure 1G). In turn, by day 21 of culture, the expression pattern resulted in a more diffuse staining localized in the cell junctions and the cytosol with both antibodies (Figure 2F). Relative quantification of mean fluorescence intensity revealed significantly increased signal using CRB1_Ab1 compared to CRB1_Ab2, while the intensity was similar on day 21 of maturation (Figure 2G). Notably, representation of the mean fluorescence profile quantified in RPE cells revealed this differential localization of CRB1 isoforms during maturation (Figure 2H).

Altogether, these results suggest that *CRB1* is expressed at different levels depending on the retinal cell type and developmental stage. For example, its total expression decreases during hiPS-RO maturation, reaching the lowest levels by D350 and resembling those found in the human retina from an elderly donor. Similarly, the abundance of *CRB1* isoforms shifts during retinal differentiation: *CRB1-A* and *CRB1-C* are predominant in immature RO whereas *CRB1-B* becomes more prominent at later stages. Additionally, our data demonstrate CRB1 expression in hiPS-RPE, where both localization and expression pattern vary according to their maturation stage.

### 2.2. Cohort of Patients Carrying Pathogenic Variants in CRB1 Causing CD, CRD, and LCA

Next, we aimed to establish genotype–phenotype correlations based on the pathogenic impact of *CRB1* variants on the three isoforms. For that purpose, we analyzed the genetic and clinical data in a cohort of 25 patients diagnosed with different IRD caused by pathogenic *CRB1* variants.

**Figure 3:**
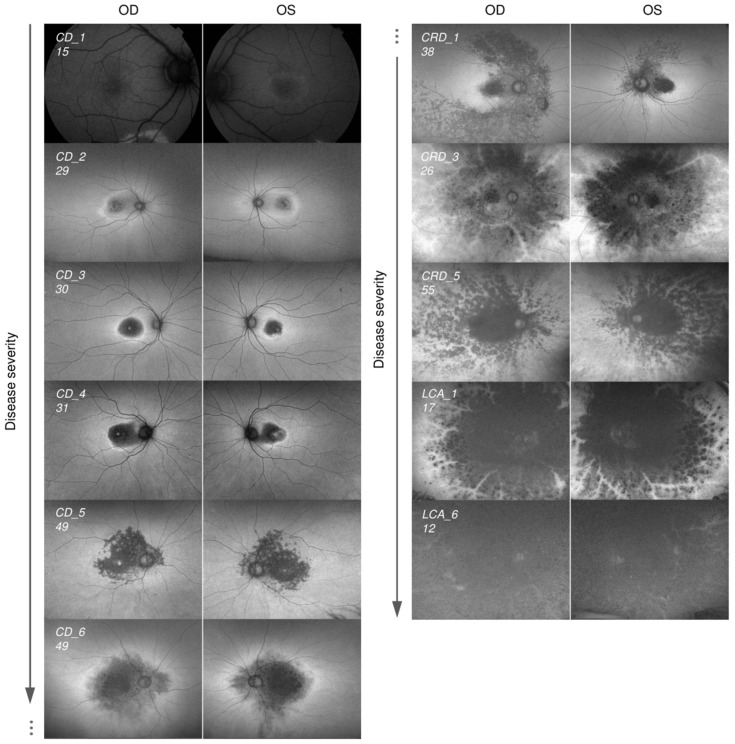
Cohort of patients carrying pathogenic variants in *CRB1* causing CD, CRD, and LCA. Fundus autofluorescence images of some patients in our cohort ordered by the degree in disease severity. OD and OS refer to right and left eye, respectively. Patient ID and age in years are indicated in each image. Abbreviations: CD, cone-dystrophy; CRD, cone-rod dystrophy; LCA, Leber congenital amaurosis.

Following routine ophthalmologic assessment—including anamnesis, visual acuity testing, slit-lamp examination, fundus examination and multimodal imaging—we classified the patients into three categories or phenotypes (Figure 3 and Table 1): (I) Cone dystrophy—Six patients were classified as CD, exhibiting predominantly macular involvement. Two cases displayed more extensive retinal alterations, with hypoautofluorescence extending to the vascular arcades; however, in both cases, rod function remained normal, as demonstrated by preserved scotopic electroretinogram (ERG) responses. These more severe CD phenotypes have also previously been described in the literature as retinitis pigmentosa inversa (characterized by central degeneration with preserved peripheral retina). To avoid confusion, we retained the CD designation for these cases [28]. (II) Cone–rod dystrophy—Nine patients were classified as CRD or related phenotypes, showing varying degrees of both macular and peripheral retinal involvement. This group also included three exhibiting specific phenotypes strongly associated with *CRB1* mutations, such as retinitis pigmentosa with preserved para-arteriolar retinal pigment epithelium (PPRPE) [31]. Other patients included in this category presented heterogeneous features, but all shared the common trait of diffuse retinal disturbances with both macular and peripheral retinal involvement. (III) Leber congenital amaurosis—Ten patients were classified as LCA based on severe early-onset retinal degeneration. Most individuals in our cohort were diagnosed before the age of six months and all before the age of two. Interestingly, four out of ten patients with LCA showed the characteristic preserved para-arteriolar RPE, a finding frequently reported in association with *CRB1* mutations [31].

We identified several reported and prevalent *CRB1* pathogenic variants, such as the in-frame deletion c.498_506delAATTGATGG (p.Ile167_Gly169del), and the missense c.2843G>A (p.Cys948Tyr), both located in EGF-like domains in exon 2 and 9, respectively. These pathogenic variants were the most prevalent in our cohort, present in 40% and 36% of our patients, respectively (Table 1). Notably, p. Ile167_Gly169del has been reported as a hypomorphic mutation, and p.Cys948Tyr as a highly deleterious variant, likely due to its impact on splicing and mRNA processing [30,38]. Importantly, all probands carrying the hypomorhpic variant c.498_506delAATTGATGG were diagnosed with the milder, macular form of IRD. Other prevalent variants found in our cohort were c.2290C>T (p.Arg764Cys) and c.613_619delATAGGAA (p.Ile205AspfsTer13) (n = 4) (Table 1).

**Table 1:** Clinical and genetic data of the patients in the cohort.

ID *	ID_2	Clinical Diagnosis	Nucleotide Change	Protein Change	Zygosis	Exon	Age of Onset		Age	BCVA OD *	BCVA OS *	Ref.
Fi25/03_01	CD_1	CD	c.498_506delAATTGATGG	p.Ile167_Gly169del	homo	2	14	adolescence	21	0.8	0.4	[39]
Fi25/04_01	CD_2	CD	c.498_506delAATTGATGG	p.Ile167_Gly169del	het	2	11	late childhood	38	0.3	0.1	[39]
			c.3299T>C	p.Ile1100Thr	het	9						[40]
Fi25/05_01	CD_3	CD	c.498_506delAATTGATGG	p.Ile167_Gly169del	homo	2	22	adult	32	0.4	0.16	[39]
Fi25/06_01	CD_4	CD	c.498_506delAATTGATGG	p.Ile167_Gly169del	het	2	13	adolescence	35	0.3	0.4	[39]
			c.3299T>G	p.Ile1100Arg	het	9						[41]
Fi25/07_01	CD_5	extensive CD/inverse RP	c.498_506delAATTGATGG	p.Ile167_Gly169del	het	2	43	adult	51	0.05	0.025	[39]
			c.1084C>T	p.Gln362Ter	het	5						[42]
Fi25/08_01	CD_6	extensive CD/inverse RP	c.498_506delAATTGATGG	p.Ile167_Gly169del	het	2	14	adolescence	61	0.04	0.04	[39]
			c.3055_3059dup	p.Met1020IlefsTer4	het	9						[43]
Fi25/09_01	CRD_1	CRD	c.498_506delAATTGATGG	p.Ile167_Gly169del	het	2	10	late childhood	48	0.1	0.1	[39]
			c.2843G>A	(Splice) p.Cys948Tyr	het	9						[44]
Fi15/29_01	CRD_2	PPRPE	c.498_506delAATTGATGG	p.Ile167_Gly169del	het	2	0	infancy	47	LP	HM	[39]
			c.2843G>A	(Splice) p.Cys948Tyr	het	9						[44]
Fi25/20_01	CRD_3	PPRPE	c.2290C>T	p.Arg764Cys	homo	7	6	late childhood	31	0.5	0.16	[44]
Fi25/20_02	CRD_4	PPRPE	c.2290C>T	p.Arg764Cys	homo	7	?		36	0.04	0.04	[44]
Fi25/10_01	CRD_5	CRD	c.498_506delAATTGATGG	p.Ile167_Gly169del	het	2	12	late childhood	58	0.05	0.05	[39]
			c.1604T>C	p.Leu535Pro	het	6						[45]
Fi25/11_01	CRD_6	CRD	c.498_506delAATTGATGG	p.Ile167_Gly169del	homo	2	<20	adolescence	64	0.06	0.06	[39]
			c.1360G>A	p.Gly454Arg	homo	6						[46]
Fi25/12_01	CRD_7	CRD	c.742T>A	p.Cys248Ser	het	3	17	adolescence	39	0.05	0.05	This study
			c.4005+1G>A	-	het	11						[47]
Fi25/13_01	CRD_8	CRD	c.407G>T	p.Cys136Phe	het	2	7	late childhood	57	LP	0.2	This study
			c.2843G>A	(Splice) p.Cys948Tyr	het	9						[44]
Fi25/14_01	CRD_9	CRD	c.1760G>A	p.Cys587Tyr	het	6	7	late childhood	67	LP	LP	[6]
			c.2843G>A	(Splice) p.Cys948Tyr	het	9						[44]
Fi25/15_01	LCA_1	LCA	c.613_619delATAGGAA	p.Ile205AspfsTer13	het	2	<2	infancy	20	0.04	0.04	[48]
			c.2290C>T	p.Arg764Cys	het	7						[44]
Fi25/15_02	LCA_2	LCA	c.613_619delATAGGAA	p.Ile205AspfsTer13	het	2	<2	infancy	15	0.04	0.05	[48]
			c.2290C>T	p.Arg764Cys	het	7						[44]
Fi15/13_01	LCA_3	LCA	c.613_619delATAGGAA	p.Ile205AspfsTer13	het	2	<2	infancy	11	0.2	0.1	[48]
			c.2843G>A	(Splice) p.Cys948Tyr	het	9						[44]
Fi25/21_01	LCA_4	LCA	c.2843G>A	(Splice) p.Cys948Tyr	homo	9	<2	infancy	23	0.05	0.05	[44]
Fi25/16_01	LCA_5	LCA	c.613_619delATAGGAA	p.Ile205AspfsTer13	homo	2	<2	infancy	46	0.01	0.04	[48]
Fi25/17_01	LCA_6	LCA	c.3280C>T	p.Gln1094Ter	homo	9	<2	infancy	16	0.016	0.025	This study
Fi25/18_01	LCA_7	LCA	c.209delT	p.Met70ArgfsTer17	het	2	<2	infancy	32	HM	HM	This study
			c.2843G>A	(Splice) p.Cys948Tyr	het	9						[44]
Fi25/19_01	LCA_8	LCA	c.2843G>A	(Splice) p.Cys948Tyr	het	9	<2	infancy	50	-	-	[44]
			c.3988G>T	p.Glu1330Ter	het	11						[45]
Fi25/22_02	LCA_9	LCA	c.2843G>A	(Splice) p.Cys948Tyr	het	9	6 months	infancy	48	HM	LP	[44]
			c.3749+2_3749+3delTG	-	het	9						[49]
Fi25/22_01	LCA_10	LCA	c.3749+2_3749+3delTG	-	homo	9	<2	infancy	70	NLP	NLP	[49]

* Abbreviations: ID, code institution/family number_patient number. BCVA, best corrected visual acuity. LP indicates light perception; NLP no light perception; HM hand movement.

Additionally, we also detected four novel pathogenic variants (Table 2). Specifically, two were missense variants (c.407G>T (p.Cys136Phe) and c.742T>A (p.Cys248Ser)), and the other two were truncating variants (c.209delT (p.Met70ArgfsTer17) and c.3280C>T (p.Gln1094Ter)). Notably, the missense mutations were allocated in residues predicted as highly likely pathogenic according to Alphaphold (Figure S3) [50]. Both substitutions involve cysteine residues that normally participate in disulfide bond formation, which is essential for stabilizing CRB1 protein structure [51]. Remarkably, the missense variants were identified in individuals with CRD, whereas the truncating variants were found in LCA probands (Table 1).

### 2.3. Impact of the Pathogenic Variants on CRB1 Isoforms Determines IRD Clinical Manifestation

Pathogenic variants can differentially impact protein expression and/or function, triggering a wide variety of disease-associated phenotypes. Given that *CRB1* has different isoforms with distinct expression patterns during retinal development, we investigated whether the localization and type of the mutations could determine the pathogenic phenotype in *CRB1*-related dystrophies. Thus, we aimed to establish a genotype–phenotype correlation considering the specific pathogenic variant, the isoform(s) affected, and the resulting clinical presentation.

**Figure 4:**
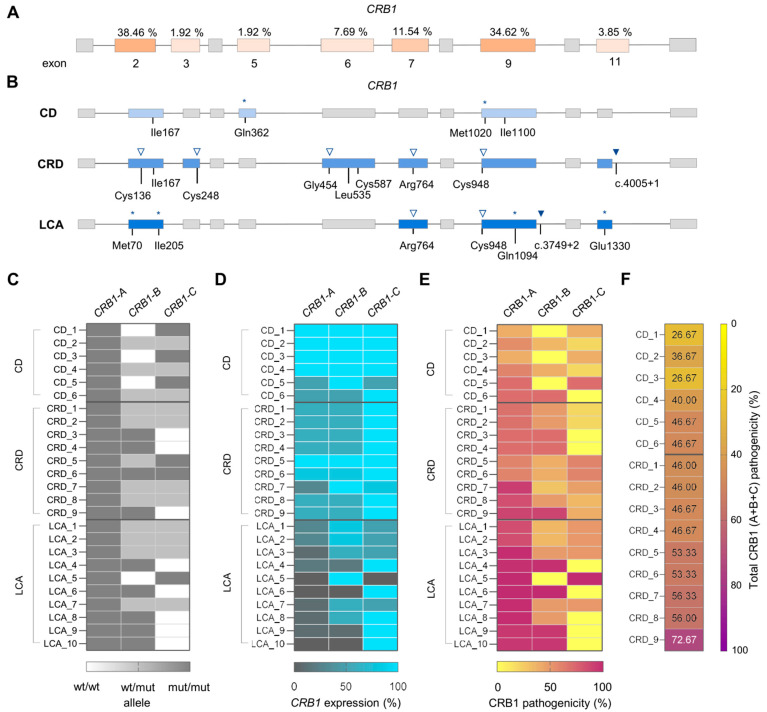
Impact of the pathogenic variants on *CRB1* isoforms determines IRD clinical manifestation. (**A**) Scheme depicting the mutation frequency in each exon in our cohort. (**B**) Representation of the variants in our cohort depending on clinical classification. Asterisks indicate truncating variants, arrowheads splice-modifying, and empty arrowheads missense mutations altering the splicing. (**C**) Heatmap representing the zygosity of each pathogenic variant per isoform. (**D**) Heatmap showing estimated *CRB1* expression depending on the mutations harbored by each patient. Expression was set as a maximum of 100% considering the sum of both alleles. (**E**) As in (**D**) but for the estimation of CRB1 pathogenicity. (**F**) Sum of total CRB1 pathogenicity calculated in (**E**) for CD/CRD patients, and expressed as total percentage.

To this end, we first examined the distribution of mutations identified across the three clinical groups. Exons 2 and 9 accounted for more than 70% of all variants, while the remaining mutations were located in exons 3, 5, 6, 7, and 11 (Figure 4A). Specifically, individuals diagnosed with CD carried pathogenic variants in exons 2 and 9, and only one proband harbored a mutation in exon 5 (Figure 4B). In contrast, mutations in individuals with CRD or LCA were distributed across exons 2, 3, 6, 7, 9, and 11 (Figure 4B). Remarkably, in our cohort, exon 6, which is the only exon shared by all three *CRB1* transcripts, was found mutated exclusively in CRD cases.

Next, we analyzed the type of pathogenic variants present in each clinical group. All CD patients carried the hypomorphic p.Ile167_Gly169del variant in homozygosis, or in heterozygosis in combination with a missense or truncating mutation in the second allele (Table 1 and Figure 4B). In CRD, the most frequent variants were also this hypomorphic in-frame deletion variant and missense mutations predicted to affect splicing, but no truncating mutations were identified (Figure 4B). Finally, all LCA patients, who present the most aggressive and early-onset phenotypes, carried truncating or splicing-modifying variants (Figure 4B).

Consistent with previous studies, *CRB1-A* was biallelically affected in all probands in our cohort, whereas *CRB1-B* and *CRB1-C* could be either mutated or not (Figure 4C) [30,33,38,52]. However, *CRB1-B* was more frequently altered in CRD/LCA patients than in the CD group (Figure 4C). To further explore the impact of the pathogenic variants in this cohort, we estimated the relative expression levels of each isoform considering the type of mutation affecting each allele. For this, we applied a scale from 0 to 100, in which 100 represented wild-type expression (i.e., mutations not affecting *CRB1* expression level, such as missense variants), and 0 indicated complete absence of mRNA or protein (as expected, for example, from two truncating variants). This estimation was conducted assuming a 50% of maximum expression per allele and mutation. Of note, probands in Figure 4C–F were ordered by clinical severity, as described in Section 4.

Examining these data, we observed that individuals with CD and CRD showed a lower overall impairment of *CRB1* expression levels compared to LCA probands (Figure 4D). Indeed, the milder the alteration in *CRB1* expression, the milder the resulting clinical phenotype. Furthermore, LCA patients exhibited an almost complete absence of *CRB1-A* expression, while *CRB1-B* was considerably compromised in some cases (Figure 4D). This observation suggests that the near-complete loss of *CRB1-A* expression may be a key determinant of the LCA condition.

Next, we sought to predict the pathogenicity levels associated with the previously estimated *CRB1* expression levels. As described in Section 4, the pathogenicity of missense and in-frame variants was assessed based on their predicted deleteriousness and their potential effects on splicing as summarized in Table 3. Accordingly, the greater the predicted damaging impact of a variant, the lower amount of functional CRB1 expected from the estimated expression.

Interestingly, we found that *CRB1-A* was affected in all cases, and its pathogenicity showed a strong association with the degree of clinical severity (Figure 4E). In fact, this analysis revealed a clear CRB1 pathogenicity in CD patients, although its expression levels were not estimated to be reduced in cases harboring missense mutations (Figure 4D,E).

Of note, *CRB1-A* was the most abundant isoform during the early stages of hiPS-RO maturation, and, intriguingly, LCA probands, who present the earliest onset among these macular dystrophies, exhibited an almost complete absence of this isoform (Figure 1E and Figure 4E) [15,21]. Therefore, these findings suggest that the lack or dysfunction of this isoform during retinal development may underlie the premature onset of LCA, linking the early developmental requirement for this abundant isoform to disease pathogenesis. Moreover, all LCA patients displayed a ≥85% loss of CRB1-A expression, in contrast to the CD and CRD groups. Conversely, the pathogenicity associated with CRB1-C did not appear to be determinant in any of the groups.

Regarding the non-congenital macular dystrophies, CRD cases exhibited overall higher pathogenicity scores than the CD group, particularly for CRB1-B, consistent with the higher proportion of splicing and damaging missense variants carried by the patients (Figure 4B,E). In an effort to identify features that could discriminate between CD and CRD, we next considered the combined pathogenicity estimates across all three isoforms and represented it as total percentage. Notably, we observed a clear correlation between total CRB1 pathogenicity and the degree of clinical severity: all CD patients showed percentages below 50%, whereas most CRD individuals exceeded this threshold (Figure 4F).

Collectively, the results obtained from our cohort indicate a strong correlation between the impact of each mutation affecting *CRB1* isoforms, the stage of retinal development at which these isoforms are required, and the resulting clinical presentation. We found that clinical phenotype is strongly conditioned by the pathogenicity in CRB1-A, in which a low impairment determined a milder macular dystrophy, whereas higher alterations were linked to LCA. Additionally, we observed that the severity of macular dystrophy correlates with the overall estimated CRB1 pathogenicity.

## 3. Discussion

Pathogenic variants in *CRB1* are responsible for several types of retinal dystrophies leading to a broad range of clinical manifestations. Consequently, there is an increasing need to establish genotype–phenotype correlations that may help to better define disease progression. Various authors have used genetically engineered mouse models, such as the *Crb1^rd8^* mouse, to study the molecular basis of *Crb1* in the murine retina. However, despite the severe retinal disorganization and disrupted outer limiting membrane (OLM) observed in this model, photoreceptors degeneration is notably absent [5]. Moreover, few studies have investigated the role and involvement of *CRB1* in the different retinal cell types, limiting our understanding of the molecular mechanisms driving the disease-associated phenotype. In that regard, hiPSCs constitute a powerful system to study *CRB1* in human-derived models [53]. Thus, in this study, we used several retinal cell models derived from hiPSCs to comprehensively evaluate *CRB1* expression in human cells, together with a cohort of 25 patients affected by different IRD carrying *CRB1* mutations to define genotype–phenotype correlations.

In the mature retina, *CRB1* exhibits two major isoforms, the canonical *CRB1-A* and *CRB1-B*, while a third transcript, *CRB1-C*, was predicted to be expressed in the human retina although at moderate levels [15,54]. Both *CRB1-A* and *CRB1-B* have been associated to photoreceptors and Müller glial cells, where *CRB1* is essential for maintaining cellular integrity [15]. Recently, Peng et al. reported its expression in the apical junctions of the murine RPE, where it plays a critical role in preserving epithelial-barrier integrity [19]. Consistently, Wang et al. reported structural and molecular defects in *CRB1*-mutant hiPS-RPE, including abnormal morphology, impaired barrier function, and phagocytosis [20]. Nevertheless, little is known about *CRB1* expression and function in human RPE and during retinal cell differentiation.

In this study, we demonstrate *CRB1* expression at both mRNA and protein level in hiPS-RPE, although total expression of *CRB1* was considerably lower compared with RO and photoreceptor-like cells. Interestingly, *CRB1-C* was the most abundant isoform in this cell-type, suggesting that this isoform may play a distinct role compared to *CRB1-A* and *CRB1-B*. We also observed that RO exhibited differential expression patterns of the three isoforms depending on the maturation stage of these 3D models. Interestingly, *CRB1-A* was initially the predominant isoform but decreased as the organoids matured, being progressively replaced by *CRB1-B*. Similarly, total *CRB1* expression declined over RO maturation, suggesting a tight regulation of CRB1 during retinal development.

In our cohort of patients, we identified four novel *CRB1* variants associated with CRD and LCA. Additionally, the in-frame deletion c.498_506delAATTGATGG located in the EGF-like fourth domain was present in all CD and some CRD cases. As previously reported, all individuals with LCA carried truncating or splice-modification mutations, consistent with the more aggressive nature of this phenotype [30]. LCA probands showed severely compromised *CRB1-A* expression, which may be a determining factor in disease development. Interestingly, *CRB1-A* was abundantly expressed during the first stages of RO maturation, in line with the early onset of LCA-related pathology. In contrast, *CRB1-C* alone did not appear to be determinant, as it was indistinctly affected across all clinical groups. Further molecular and biochemical studies will be critical to elucidate CRB1 function in retinal cells, provide insight into the physiology of vision, and help define the events that lead to blindness.

Patients with CD, who exhibit a milder phenotype, showed overall lower levels of CRB1 pathogenicity compared to CRD and LCA patients. Analysis of CRB1-pathogenicity suggested that CRB1-B levels could be particularly relevant for determining disease severity within the non-congenital macular dystrophies, in which lower levels of pathogenic CRB1 were associated with CD patients. Strikingly, calculation of total CRB1 pathogenicity positively correlated with the degree of clinical severity between these two clinical groups, CD and CRD.

The clinical heterogeneity triggered by *CRB1* mutations underscores the complexity of linking pathogenic variants with disease manifestation. Depending on the location and type of pathogenic variant per se, *CRB1* mutations may differentially affect *CRB1-A* and *CRB1-B*, thereby contributing to phenotypic severity and determining whether patients develop non-congenital macular dystrophy or LCA. This is also a key factor to consider in order to design targeted therapies for the CRB1-related retinopathies. As the results presented here demonstrate, the expression levels of the different isoforms fluctuate, not only during development, but also depending on the maturation stage. Hence, understanding the precise pattern of *CRB1* expression in each case is of outmost importance for the optimization of gene therapies and other potential treatment strategies.

Collectively, our findings provide new insights into the genotype–phenotype correlations in CRB1-related retinal dystrophies and suggest that disease severity is linked to the impact of mutations on the isoforms expression. Moreover, the differential expression patterns of *CRB1* transcripts during retinal development highlight a close relationship between the specific isoform affected and the resulting phenotype. Studying the different isoforms encoded by *CRB1* during cellular differentiation and maturation proves to be essential, not only to advance the understanding of IRD pathophysiology, but also for designing effective and optimal personalized medicine.

## 4. Materials and Methods

### 4.1. Patients and Clinical Evaluation

A total of 25 patients were clinically diagnosed at the Institut de Microcirurgia Ocular (Barcelona, Spain) based on standard ophthalmic evaluations. Most patients were evaluated by multimodal imaging (retinography, autofluorescence retinography, and optical coherence tomography), and when in doubt about the degree of retinal involvement, other testing such as electroretinogram (ERG) or visual field (VF) was also performed. Patients were sub-classified into three different categories depending on the degree/location of the retinal disturbances: (I) CD and related phenotypes. This category included those cases with predominantly macular involvement, with preserved periphery and rod-function. Dubious cases were tested with VF and ERG and were classified into this category when peripheral VF was normal and/or the scotopic responses on the ERG were preserved or only minimally affected. (II) CRD and related phenotypes. Patients included in this category showed both macular and peripheral involvement in variable degrees, but the peripheral involvement had to be severe enough as to affect the VF and/or scotopic ERG. (III) LCA. This category included patients that complied with the generally accepted diagnostic criteria for LCA: early-onset retinal dystrophy (usually diagnosed shortly after birth or within the first two years), responsible for congenital blindness and with associated signs, such as nystagmus, abnormal pupillary responses, and severely diminished or abolished responses on ERG [55]. The study was conducted in accordance with the Declaration of Helsinki and was approved by the Ethics Committee of the Institut de Microcirurgia Ocular (Protocol code: 170505_117. Date of approval: 2 June 2017).

### 4.2. Next-Generation Sequencing and Identification of Pathogenic Variants

Peripheral blood was collected from patients and relatives in tubes containing EDTA. Automated extraction of genomic DNA was performed using the KingFisher Duo purification system (Thermo Fisher Scientific, Waltham, MA, USA) for genetic analyses. All procedures were in accordance with the Declaration of Helsinki. Ethics approval was received from the Ethics Committee of IMO (160321_96). All probands were fully informed of the purpose and procedures of this study, and a written informed consent was obtained from each individual.

WES was performed in nine probands by Macrogen (Madrid, Spain) using libraries designed and constructed with the SureSelect V5 or V6 technology (Agilent, Santa Clara, CA, USA), and generated amplicons were genotyped with the HiSeq 3000 or 4000 (Illumina, San Diego, CA, USA). The FASTQ raw data files obtained were analyzed using the GeneSystems online platform (Sistemas Genómicos, Valencia, Spain), aligning sequences against the reference genome GRCh38/hg38. WES results were filtered to look at the genes included in this IRD panel. The detected variants were then considered according to the deleterious potential, the familial inheritance hypothesized and the minor allele frequency (MAF ≤ 0.001), from the following open databases: 1000 Genomes, ExAC, GO ESP, TOPMed, and EVS. Predictions on pathogenicity and splicing modifications were performed in silico using various tools. Missense mutations were analyzed with ENSEMBL Variant Effect Predictor (VEP), which provides results from a range of algorithms to assess the potential pathogenicity of a variant. Predictors used by VEP were SIFT, PolyPhen, AlphaMissense, BayesDel_addAF, DEOGEN2, ESM1b, LIST-S2, MetaLR, MetaRNN, MetaSVM, MutationAssessor, MutationTaster, PROVEAN, PrimateAI, and fathmm-XF_coding. Predictions of in-frame variants were MutationTaster2025, SIFT_indels2, and MutPred-Indel.

ALAMUT software (version 1.4; Sophia Genetics, Rolle, Switzerland) was used for splicing predictions, which uses the following bioinformatic tools: Splice Site Analysis (SFF), MaxEnt, Splice Site Prediction by Neural Network (NNSPLICE), and GeneSplicer. All the resulting variants were contrasted with the mutation databases HGMD and Uniprot [26,56]. Alphaphold was used for 3D structures modeling and to predict pathogenicity [50]. Putative pathogenic variants were confirmed by Sanger sequencing, and mutation segregation analyses were carried out in all cases.

### 4.3. Estimation of CRB1 Mutations Impact on Pathogenicity

Estimation of the pathogenic variant impact on *CRB1* expression (Figure 4D) was performed by subtracting a percentage of expression from the 50% corresponding to one of the two alleles. Thus, an unaffected allele is considered as the 50% of expression. Truncating variants were considered as 0% of expression, while in-frame deletions and missense variants as the 50% (not altered). For mutations in splice-sites (+1 to +3) the remaining 5% of potential wild-type transcripts was considered.

The estimation of the impact on CRB1 pathogenicity (Figure 4E) was performed from the amount of expression calculated and was modified while taking into account the potential impact of in-frame and missense variants. The in-frame pathogenic variant impact was set as a 20% reduction. Missense-derived pathogenicity was quantified considering the damaging and alteration of the canonical splicing as specified in Table 3. Specifically, damaging predictions were performed as follows: 6–10D 50%; 11–12D 70%; 13–14D; 80%. Probands in heatmaps and Tables appeared in the same order that was set according to ophthalmologist’s criteria of clinical severity, from least to most affected.

### 4.4. Human iPSCs Culture and Differentiation into Retinal Cell Models

Human iPSCs were obtained as previously described from individuals without any ophthalmologic disease and no genetic variants related to retinal dystrophies [57]. Briefly, hiPSC colonies were maintained in StemFlex medium (Thermo Fisher Scientific, Waltham, MA, USA) and cultured on Matrigel-coated dishes (Merck, Bedford, MA, USA). hiPSCs were differentiated into RPE, photoreceptor-like cells, and RO as previously described [58,59,60]. Photoreceptor-like progenitors were generated by culturing cells in Retinal induction media followed by neural differentiation media supplemented with LDN-193189 (Reprocell, Yokohama, Japan). Cells were then scrapped and plated into ultra-low attachment plates to form spheres that were plated and cultured without LDN-193189 until formation of photoreceptor-like cells. RPE cells were differentiated by culturing hiPSCs in the presence of 10 mM Nicotinamide (Merck, Bedford, MA, USA), 100 ng/mL Activin A (Stem Cell Technologies, Vancouver, BC, Canada), or 3 µM CHIR99021 (Merck). Retinal organoids were formed from NR arising in cultures incubated in proneural induction media. Optical vesicles were excised and cultured in low-binding 96-multiwell plates in the presence of retinal maturation mediums supplemented or not with retinoic acid. Cells were visualized using a Zeiss Axiovert microscope or EVOS XL Core cell Imaging system (Thermo Fisher Scientific).

### 4.5. PCR Amplification and Sanger Sequencing

PCR amplification of the desired genomic region was performed and run on a gel to examine the resulting bands and size using different primer pairs, as specified in Figure Legends. PCR products of the desired genomic region were purified using 96-well Acroprep Advance plates (Pall Corporation, Ann Arbor, MI, USA) with a vacuum manifold (Pall Corporation), or using a GeneJET PCR purification kit (Thermo Fisher Scientific) after band isolation, and Sanger sequenced (Macrogen).

### 4.6. Immunofluorescence and Image Analysis

For immunofluorescence analysis, hiPS-RO were fixed in 4% paraformaldehyde (PFA) (Thermo Fisher Scientific) at 4 °C for 1 h, incubated in 30% sucrose in PBS at 4 °C overnight, and embedded in O.C.T. mounting media (VWR). A total of 12 µm cryosections were air-dried, permeabilized with 0.25% Triton X-100 in PBS, and incubated for 1 h at room temperature in blocking solution (5% fetal bovine serum, 4% bovine serum albumin, and 0.5% Tween-20 in PBS). hiPS-RPE and hiPS-photoreceptor cultures were fixed in 4% PFA and blocked for 1 h at room temperature. Slides and cells were then incubated with primary and secondary antibodies listed in Table S1 at 4 °C overnight or at room temperature, respectively. Cuts were counterstained with DAPI (Thermo Fisher Scientific), visualized with Zeiss Axiovert and Axiocam 503 mono (Carl Zeiss Inc., Jena, Germany), and analyzed with ImageJ software 1.53k (NIH, Bethesda, MD, USA). At least three different samples were used for evaluating each staining.

### 4.7. RNA Extraction and Real-Time PCR

RNA from hiPSC-derived retinal cells was extracted using TRIzol (Thermo Fisher Scientific), as per manufacturer’s instructions. cDNA synthesis was conducted using Transcriptor First Strand cDNA Synthesis Kit (Roche Diagnostics, Basel, Switzerland), and quantified by real-time PCR using QuantStudio™ and specific primers for SybrGreen detection (Thermo Fisher Scientific) listed in Table S2. TaqMan probes (Thermo Fisher Scientific) were used for quantification of *BEST1*, *RCVRN*, *RLBP1*, and *CRB1-A* (exons 11 and 12). Commercially available cDNA of the retina from a 77-year-old human donor was obtained from BioChain Institute Inc. (Newark, CA, USA). Prism 10.1.2 was used for data representation (GraphPad Software, La Jolla, CA, USA).

## 5. Conclusions

This study defines the precise expression pattern of the three *CRB1* isoforms (*CRB1-A, CRB1-B*, and *CRB1-C*) in the different cell-types of human retinal models. Moreover, this work demonstrates that the relative levels of *CRB1* isoforms fluctuate depending on the developmental and maturation stage. Additionally, the results derived from the analysis of a cohort of 25 patients carrying pathogenic *CRB1* variants allowed us to stablish a genotype–phenotype correlation. Actually, the pathogenic impact of each mutation is conditioned by the affectation of each isoform, and, notably, it positively correlates with the patient’s clinical severity.

## Figures and Tables

**Table 2:** In silico analysis of new variants pathogenicity.

nt Change	Protein Change	dbSNP ^a^	MAF ^b^	Predictors ^c^
c.209delT	p.Met70ArgfsTer17	Not referenced	Not referenced	truncating
c.407G>T	p.Cys136Phe	rs752559648	Not referenced	12D
c.742T>A	p.Cys248Ser	rs769980214	A = 0.000002 (1/595,670)	14D
c.3280C>T	p.Gln1094Ter	Not referenced	Not referenced	truncating

^a^ dbSNP, single nucleotide polymorphism database. ^b^ MAF, minor allele frequency. ^c^ Predictions are expressed as “D” for damaging.

**Table 3:** Missense variants pathogenicity and splicing prediction.

		Missense Impact		Splice Impact	
Variant ^a^	Protein Change	Predictors ^b^	Pathogenicity Estimation ^c^	Predictors ^b^	Pathogenicity Estimation ^d^
* c.407G>T	p.Cys136Phe	12D	70%	1G	20%
* c.742T>A	p.Cys248Ser	14D	80%	2G	40%
c.1360G>A	p.Gly454Arg	14D	80%	2G	40%
c.1604T>C	p.Leu535Pro	14D	80%	--	--
c.1760G>A	p.Cys587Tyr	13D	80%	--	--
c.2290C>T	p.Arg764Cys	6D	50%	2G	40%
c.2843G>A	p.Cys948Tyr	14D	80%	4L	80%
c.3299T>C	p.Ile1100Thr	11D	70%	--	--
c.3299T>G	p.Ile1100Arg	14D	80%	--	--

^a^ Asterisks indicate new missense variants reported in this study. ^b^ Predictions are expressed as D: damaging, G: gain, and L: loss. ^c^ Estimation of the degree of pathogenicity for each variant, that is subtracted from 50% (one allele). See Section 4 for details. ^d^ Estimation of the degree of pathogenicity for each variant, that is subtracted from the estimated CRB1 expression. See Section 4 for details.

## Data Availability

The original contributions presented in this study are included in the article/Supplementary Material. Further inquiries can be directed to the corresponding author.

## Supplementary Materials

The following supporting information can be downloaded at https://www.mdpi.com/article/10.3390/ijms262311551/s1.
