# Supplementary material for: The Specific Pathogenicity Pattern of the Different CRB1 Isoforms Conditions Clinical Severity in Inherited Retinal Dystrophies
Source: Int J Mol Sci. 2025 Nov 28;26(23):11551. doi: 10.3390/ijms262311551 (PMC12692272; doi:10.3390/ijms262311551)
Supplement: Supplementary file 1 [file ijms-26-11551-s001.zip › ijms-3974238-supplementary.pdf]

Figure S1

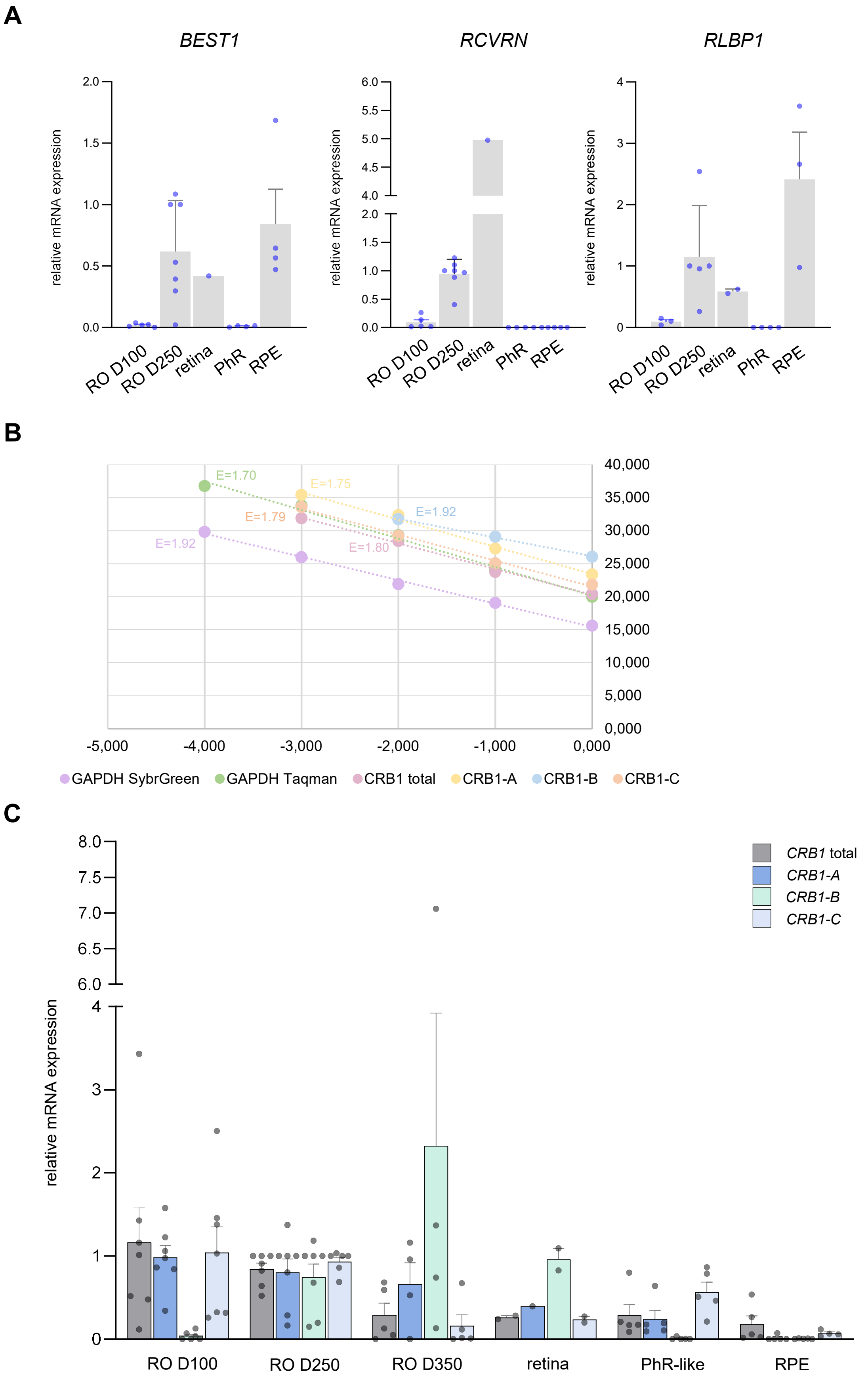

Figure S1. (A) Relative mRNA quantification of retinal cells markers in the different models. **(B)** Linear regressions of the primer pairs used for qPCR. **(C)** As in (A) but detecting total *CRB1* and the three isoforms, and analyzed using the Pfaffl method. Abbreviations: RO, retinal organoid; PhR, photoreceptor; RPE, retinal pigment epithelium.

Figure S2

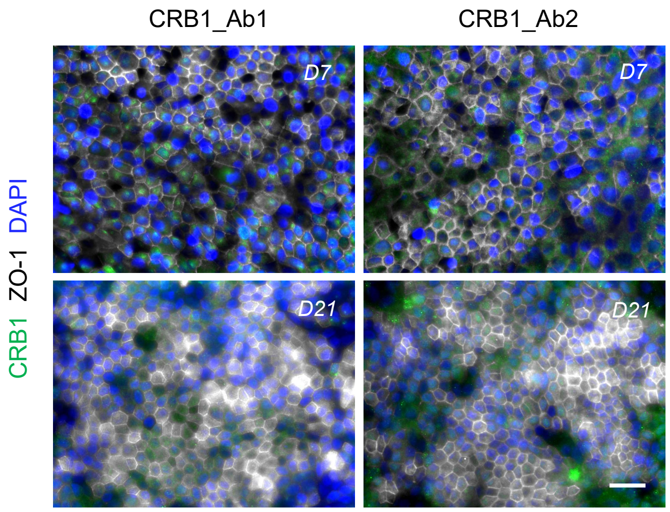

Figure S2. Pictures in Figure 2F showing CRB1 and ZO-1 counterstained with DAPI. Scale bar represents 25 μm.

Figure S3

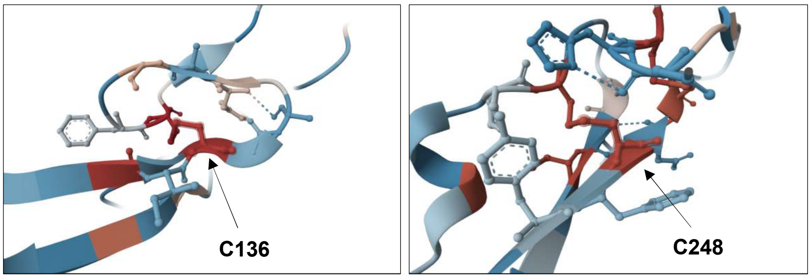

Figure S3. 3D CRB1 structures predicted with Alphaphold showing the residues of the two new missense variants reported in this study. Nucleotides in red indicated residues with predicted likely pathogenic variants.

Table S1. List of antibodies

Antibody	Source	ID
Rabbit ploylonal Arrestin 3	Thermo Fisher Scientific	PA5-114476
Mouse monoclonal CRALBP	Abcam	ab15051
Rabbit ploylonal CRB1 (CRB1_Ab1)	Proteintech	26598-1-AP
Rabbit ploylonal CRB1 (CRB1_Ab2)	Thermo Fisher Scientific	PA5-143856
Rabbit polyclonal anti-Recoverin	Affinity	DF3160
Mouse monoclonal anti-Rhodopsin	Thermo Fisher Scientific	MA1-722 RRID:AB_325050
Mouse monoclonal anti-RPE65	Thermo Fisher Scientific	401.8B11.3D9
Mouse monoclonal anti-ZO-1	Thermo Fisher Scientific	ZO1-1A12 RRID:AB_2533147
Goat anti-rabbit Alexa Fluor-568	Thermo Fisher Scientific	A11036
Goat anti-rabbit Alexa Fluor-488	Thermo Fisher Scientific	A32731
Donkey anti-mouse Alexa Fluor-555	Thermo Fisher Scientific	A32773
Donkey anti-mouse Alexa Fluor-488	Thermo Fisher Scientific	A21202

Table S2. Primers used for qPCR

Gene/ID	Forward
CRB1 total 6F	CCAGTGGGAATGACCAGCAAT
CRB1 total R	GTTACCAGTGTGACTGCCACA
CRB1 5altF	TTTGGAGCCAGGACACATGGT
CRB1 6R	GAACATGCATCCCTCACTTCCA
CRB1 6altR	CCCCACAAGACTTCTGCTGC
GAPDH Fw	TGCGGCGCCATCTGCCCG
GAPDH Rv	GCCGGCGCTGCAGGAAGG
